# Targeting the Stress-Induced Protein NUPR1 to Treat Pancreatic Adenocarcinoma

**DOI:** 10.3390/cells8111453

**Published:** 2019-11-17

**Authors:** Patricia Santofimia-Castaño, Yi Xia, Ling Peng, Adrián Velázquez-Campoy, Olga Abián, Wenjun Lan, Gwen Lomberk, Raul Urrutia, Bruno Rizzuti, Philippe Soubeyran, José Luis Neira, Juan Iovanna

**Affiliations:** 1 Centre de Recherche en Cancérologie de Marseille (CRCM), INSERM U1068, CNRS UMR 7258, Aix-Marseille Université, CEDEX, 13288 Marseille, France; patricia.santofimia@inserm.fr (P.S.-C.); wenjun.lan@etu.univ-amu.fr (W.L.); philippe.soubeyran@inserm.fr (P.S.); 2 Institut Paoli-Calmettes, Parc Scientifique et Technologique de Luminy, CEDEX, 13288 Marseille, France; 3 Chongqing Key Laboratory of Natural Product Synthesis and Drug Research, School of Pharmaceutical Sciences, Chongqing University, Chongqing 401331, China; yixia@cqu.edu.cn; 4 Aix-Marseille Université, CNRS, Centre Interdisciplinaire de Nanoscience de Marseille, UMR 7325, «Equipe Labellisée Ligue Contre le Cancer», Parc Scientifique et Technologique de Luminy, CEDEX, 13288 Marseille, France; ling.peng@univ-amu.fr; 5 Instituto de Biocomputación y Física de Sistemas Complejos, Joint Units IQFR-CSIC-BIFI, and GBsC-CSIC-BIFI, 50009 Universidad de Zaragoza, Spain; adrianvc@unizar.es (A.V.-C.); oabifra@unizar.es (O.A.); jlneira@umh.es (J.L.N.); 6 Aragon Institute for Health Research (IIS Aragon), Universidad de Zaragoza, 50009 Zaragoza, Spain; 7 Centro de Investigación Biomédica en Red en el Área Temática de Enfermedades Hepáticas y Digestivas (CIBERehd), 28029 Madrid, Spain; 8 Departamento de Bioquímica y Biología Molecular y Celular, Universidad de Zaragoza, 50009 Zaragoza, Spain; 9 Fundacion ARAID, Government of Aragon, Universidad de Zaragoza, 50018 Zaragoza, Spain; 10 Instituto Aragonés de Ciencias de la Salud (IACS), Universidad de Zaragoza, 50009 Zaragoza, Spain; 11 Division of Research, Department of Surgery and the Genomic Sciences and Precision Medicine Center (GSPMC), Medical College of Wisconsin, Milwaukee, WI 53226, USA; glomberk@mcw.edu (G.L.); rurrutia@mcw.edu (R.U.); 12 CNR-NANOTEC, Licryl-UOS Cosenza and CEMIF.Cal, Department of Physics, University of Calabria, 87036 Cosenza, Italy; bruno.rizzuti@fis.unical.it; 13 Instituto de Biología Molecular y Celular, Universidad Miguel Hernández, Edificio Torregaitán, 03202 Elche, Alicante, Spain

**Keywords:** drug design, intrinsically disordered protein, pancreatic ductal adenocarcinoma, molecular dynamics, NUPR1, stress response, spectroscopy

## Abstract

Cancer cells activate stress-response mechanisms to adapt themselves to a variety of stressful conditions. Among these protective mechanisms, those controlled by the stress-induced nuclear protein 1 (NUPR1) belong to the most conserved ones. NUPR1 is an 82-residue-long, monomeric, basic and intrinsically disordered protein (IDP), which was found to be invariably overexpressed in some, if not all, cancer tissues. Remarkably, we and others have previously showed that genetic inactivation of the *Nupr1* gene antagonizes the growth of pancreatic cancer as well as several other tumors. With the use of a multidisciplinary strategy by combining biophysical, biochemical, bioinformatic, and biological approaches, a trifluoperazine-derived compound, named ZZW-115, has been identified as an inhibitor of the NUPR1 functions. The anticancer activity of the ZZW-115 was first validated on a large panel of cancer cells. Furthermore, ZZW-115 produced a dose-dependent tumor regression of the tumor size in xenografted mice. Mechanistically, we have demonstrated that NUPR1 binds to several importins. Because ZZW-115 binds NUPR1 through the region around the amino acid Thr68, which is located into the nuclear location signal (NLS) region of the protein, we demonstrated that treatment with ZZW-115 inhibits completely the translocation of NUPR1 from the cytoplasm to the nucleus by competing with importins.

## 1. Introduction

In this short review, we summarize our 20-year-long work on NUPR1, from its cloning to the development of drugs capable of interfering with the functions of the protein. We first describe how NUPR1 is a stress-protein expressed in several tissues, and which is disordered; that is, it does not have a well-fixed secondary and tertiary structure. Thus, designing drugs against such a protein is challenging. In the second section of this review we shall describe how we have surmounted those difficulties by using a multidisciplinary approach involving several fields of expertise to repurpose a drug targeting NUPR1. Finally, we describe the mechanism of function of such a drug at the molecular level: hampering the transit of NUPR1 into the nucleus by competition with importins.

## 2. Why Is the Stress Response Essential to Cancer Cells and Why Could It Be an Exploitable Therapeutic Route?

**Figure 1:**
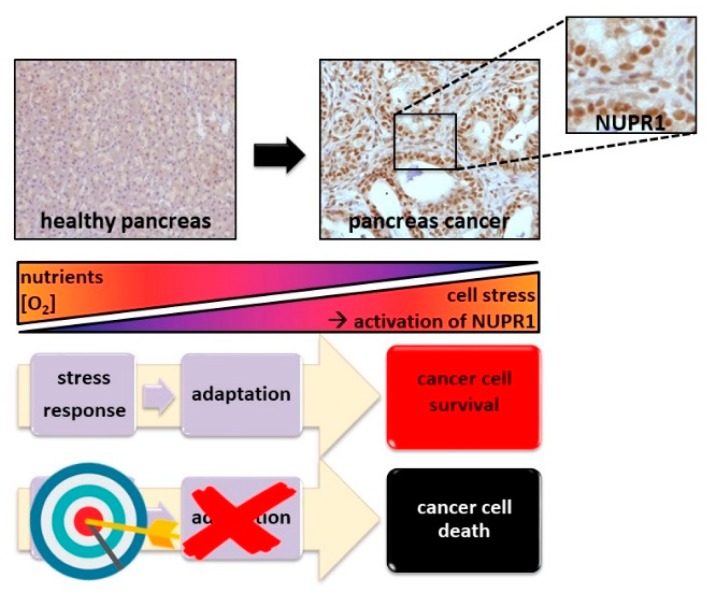
Targeting the stress response protein NUPR1 is a promising therapy for treating cancer. Pancreatic cancer cells are extremely challenged by a stressful environment due to a poor concentration of nutrient and oxygen, among other factors. In order to adapt themselves and survive, tumor cells activate stress response pathways overexpressing stress proteins such as NUPR1, which is constantly present in the tumor tissue. Our strategy is focused on targeting NUPR1 to induce cancer cell death as a therapeutic treatment for cancer.

Similar to plants, bacteria, yeast, and other uni- or multi-cellular organisms that have developed pathways to respond to environmental harsh conditions, cancer cells have developed molecular mechanisms to facilitate adaptation to a variety of stressful conditions. Cellular stress responses represent a range of molecular changes activated when a cell faces hostile environmental and cellular conditions. Cancer cells in general, and pancreatic ductal adenocarcinoma (PDAC) in particular, grow under extremely harsh circumstances. These stresses originates from: (1) the environmental conditions (i.e., hypo-vascularization with hypoxia and low contribution of nutrients, mechanical pressure, DNA-damaging agents); or (2) the cancerization processes themselves as a consequence of the altered metabolism of the transformed cells (i.e., high reactive oxygen species (ROS ) production, endoplasmic reticulum (ER) stress) [1,2], limiting their normal development and promoting a dramatic reprogramming of their phenotype [3,4,5]. These situations activate the expression of some stress proteins [6,7,8] to allow the cells to survive, grow, and ultimately progress as a tumor. Thus, cancer cells become highly dependent on the stress protein functions, and then we can assume that targeting specifically these stress factors and understanding their regulation mechanisms could be an efficient (and alternative) therapeutic cancer strategy, as already suggested years ago [9]. Among these survival mechanisms, those controlled by the stress induced protein NUPR1 seem to be the some of the most common, and therefore, promising to target (Figure 1).

## 3. The Intrinsically Disordered Stress Protein NUPR1 in PDAC

NUPR1 was first described as being activated during the acute phase of the pancreatitis [10]. Afterwards, the transitory expression of NUPR1 was discovered to be a surrogate to the stress response caused by many stimuli (including minimal stimuli) in most cell types, characterizing NUPR1 as a typical stress-associated protein [11,12]. Then, NUPR1 was found to be systematically in most of cancer tissues. At cellular level, NUPR1 was described to participate in many cancer-associated processes including cell-cycle regulation, apoptosis [13,14], senescence [15], cell migration and invasion [16], and development of metastases [17]. Indeed, NUPR1 has recently elicited significant attention due to its role in promoting cancer development and progression in pancreas [18,19]. NUPR1-dependent effects also mediate resistance to anticancer drugs [20,21,22]. Remarkably, we have previously shown that genetic inactivation of Nupr1 antagonizes the growth of pancreatic cancer [16,23], and other laboratories have also demonstrated that genetic inactivation of NUPR1 stops the growth of hepatocarcinoma [24], non-small lung cancer [25], cholangiocarcinoma [26], glioblastoma [27], multiple myeloma [28,29], and osteosarcoma [30], thereby supporting NUPR1 as a promising therapeutic target for the development of new therapies against cancers. However, genetic approaches, such as antisense oligonucleotide (ASOs)- or siRNA-based inactivation, are still far away from being used in clinic in the next few years. Therefore, we have developed an original strategy to select small compounds against NUPR1 to be used for treating PDAC.

## 4. Screening Small Compounds as Anticancer Agents against NUPR1

Structurally, NUPR1 is an 82-residue-long intrinsically disordered protein (IDP) [31,32,33,34,35]. Therefore, the current target-based high-throughput screening for drug-selection, used for well-folded proteins, is challenging for NUPR1. In general, drug-targeting IDPs is difficult due to their extremely dynamic nature [36], the typically weak binding affinities towards their natural partners, and the fact that many of them have usually several binding hotspots (all of which are features occurring in NUPR1). We have recently developed a bottom-up approach by using biophysical, biochemical, bioinformatic, and biological techniques for a molecular screening in vitro, in vivo, in silico, and in cellulo to select potential drug candidates against NUPR1 [37]. We have first characterized the interactions between NUPR1 and several potential ligands by screening a collection of 1120 compounds approved by the Food Drug Administration (Prestwick Chemical Library, http://www.prestwickchemical.com/libraries-screening-lib-pcl.html). We have used fluorescence thermal denaturation, on the basis of the largest shifts in thermal denaturation midpoints of the thermal curves of NUPR1 with an external dye in the presence and absence of the compounds of the library. In parallel, we have carried out a four-part strategy based on experimental and computational methods: (1) we have determined the thermodynamic parameters of the binding reaction with NUPR1 of the most promising compounds (i.e., those showing the largest changes in thermal shifts when compared to isolated NUPR1) by using isothermal titration calorimetry (ITC); (2) we have performed molecular dynamics (MD) simulations to obtain an ensemble of NUPR1 conformations in isolation; (3) we have used this ensemble to dock the most promising screened compounds; and (4) we have determined structure-activity relationships (SAR) by NMR with the complexes of NUPR1 and the selected compounds (i.e., detecting NUPR1 residues affected by the presence of the corresponding ligand). The blind strategy combining SAR-NMR and MD simulations validated our approach, as we have essentially observed a close analogy between the residues in contact with the compound and those residues whose signals in NMR spectra were affected by the presence of the compound (and then, when binding was happening). The dissociation constants for the compounds measured by ITC were in the same order (micromolar range) as those found for the natural binding partners of NUPR1 [14,31,32,34,35].

At the end of this procedure, we identified trifluoperazine (TFP), and its structurally related fluphenazine hydrochloride, as the compounds with the largest affinity for NUPR1. Phenotypic assays have been carried out to assess the potential bioactivity of TFP. Cell viability assays in the presence of TFP have led to half-maximal inhibitory concentration (IC_50_ values) of ~10 µM. Most importantly, tests performed with TFP in vivo, with human pancreatic cancer cell-derived xenografts implanted into immunocompromised mice, have shown an arrest of the tumor growth in a dose-dependent manner [37]. Therefore, we have successfully repurposed TFP as a possible cancer drug for treating PDAC. Unfortunately, high doses of TFP as necessary for treating PDAC have also led to unwanted neurological effects such as strong lethargy and hunched posture in the treated mice [37]. Thus, although relatively efficient as an anticancer agent, the neurological effects observed preclude the use of TFP in clinic to treat cancers.

## 5. ZZW-115 Is a Strongly Improved Trifluoperazine-Derived Compound with a New Mechanism of Action

We have developed a multidisciplinary approach to improve the efficiency of TFP by: (1) increasing its anticancer effect as much as possible with the aim of decreasing the doses to treat patients; and (2) reducing its undesirable neurological side-effects.

A rational, in silico ligand-based design relying on a combination of MD and docking guided the first steps of the organic syntheses of TFP-derived compounds. These new molecules show: (1) a stronger affinity in vitro for NUPR1 than TFP, as indicated by a combination of spectroscopic (fluorescence, NMR, and circular dichroism (CD)) and biophysical studies (ITC); and (2) the same NUPR1 binding regions as TFP.

The anticancer activity of the ZZW-115, one of the synthesized compounds, was tested on a panel of 11 primary PDAC-derived cells and it was found to be efficient to kill the cancer cells with IC_50_ in the range from 0.84 μM (ANOR cells) to 4.93 μM (HN14 cells); these in vitro results are in good agreement with the results from ITC measurements. Most importantly, ZZW-115 shows a dose-dependent tumor regression in xenografted mice leading to almost a disappearance after 30 days of treatment with 5 mg/kg/day, in four independent PDAC models [38]. Noteworthy, this happened with no apparent neurological effects.

To further show that ZZW-115 displays its anticancer activity via targeting NUPR1, we have obtained a few clones in which *Nupr1* has been inactivated by a CRISPR/Cas9 approach. As expected, we have found that three *Nupr1* KO clones are significantly more resistant to ZZW-115-treatment than two *Nupr1* WT clones. These results indicate that ZZW-115 is certainly exerting its effect by binding to NUPR1. However, these findings do not unambiguously prove that NUPR1 is the sole protein targeted by ZZW-115; rather, our results show that targeting NUPR1 seems to be the main mode of action of ZZW-115, and its binding to the protein is mainly responsible for its antitumor effect [38].

Since resistance to chemotherapy is a common issue that oncologists must face in the treatment of patients with PDAC, we have used the MiaPaCa-2 cell line, which has become resistant to the two most frequently used chemotherapeutic agents, oxaliplatin or gemcitabine, to assess whether resistance to them is also conferring resistance to ZZW-115. Remarkably, ZZW-115-treatment of resistant MiaPaCa-2 cells shows the same sensitivity as the parental cells, suggesting that the antitumor effect of the ZZW-115 is not affected by the resistance to others drugs and may act on the tumor by following some other different intracellular pathways [38].

## 6. ZZW-115 Induces Tumor Cell Death by Necroptosis and Apoptosis

At the cellular level, we have demonstrated that ZZW-115 induces cell death by both necroptotic (as measured by l-lactate dehydrogenase (LDH) release) and apoptotic (as measured by caspase 3/7 activity) mechanisms. Moreover, we have performed rescue experiments by using Necrostatin-1 and Z-VAD-FMK, either alone or in combination. Both inhibitors improved cell viability when administered alone, with a greater effect when they were used in combination. From the therapeutic point of view, the fact that ZZW-115 fosters different cell death pathways is an advantage, compared with other drugs commonly used in clinic. In fact, by using concentrations of ZZW-115 or paclitaxel (a classical pro-apoptotic drug) that induced similar caspase activation level, ZZW-115 demonstrated stronger anticancer activity. In addition, the use of a compound like ZZW-115 that is capable of promoting cell death by apoptosis, and concomitantly also necroptosis, represents the best strategy against cancers with intrinsic or acquired resistance to apoptosis (unpublished results).

**Figure 2:**
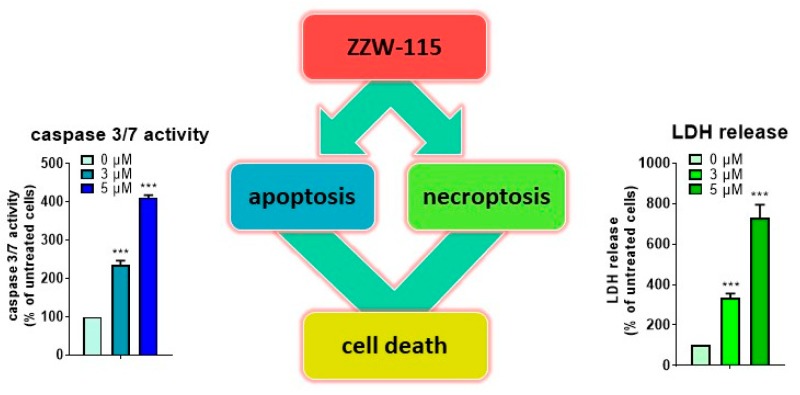
ZZW-115 is a promising therapeutic agent inducing tumor cell death by necroptosis and apoptosis. Treatment with ZZW-115, at a concentration of 3 or 5 μM for 24 h, of pancreatic cancer cells (MiaPaCa-2 cells) demonstrated that our compound was able to induce cell death by apoptosis and necroptosis on a dose-dependent manner, by measuring caspase 3/7 activity or LDH release, respectively.

It is well-known that ATP plays an important role in cell death fate. Interestingly, ZZW-115 induced a dramatic decrease of ATP content in treated cells. To better understand the causes that led to this decrease of the ATP level, we carefully studied the kinetics of the main sources of its production: oxidative phosphorylation (OXPHOS) and anaerobic glycolysis. On the one hand, OXPHOS metabolism suffered a time-dependent decrease after ZZW-115 treatment, with a great failure in mitochondrial respiration and ATP production. On the other hand, the glycolytic pathway shifted at earlier time (4 h), as an attempt to compensate the mitochondrial collapse. However, this switch to a higher glycolytic metabolism was transitory and the treated cell rapidly consumed the glycolytic reserve. As a consequence, total ATP production and content rapidly dropped after 24 h of treatment. It is well-known that disruption of mitochondrial function is a key event that triggers cell death, in which mitochondrial ROS formation has an active role. In this regard, ZZW-115 was also capable of increasing the formation of superoxide ions in the mitochondria, contributing in this way to the mitochondrial failure and cell death [38] (Figure 2). Importantly, the molecular consequences described above, which led to necroptotic and apoptotic cell death, were similar to those observed in NUPR1-deficient cells [39]. Consequently, ZZW-115 constitutes a promising drug candidate for pancreatic cancer with an original molecular mechanism, since it combines the concomitant induction of necroptosis and apoptosis with a concomitant mitochondrial failure.

## 7. ZZW-115 Is Active in Some Type of Cancers

Since NUPR1 is overexpressed in several (if not all) tumors, we have evaluated the effect of treating cellular lines derived from several tumors with increasing concentrations of ZZW-115. Treatment of cells such as U87 (glioblastoma), A375 and B16 (melanoma), U2OS and SaOS-2 (osteosarcoma), HT29, SK-CO-1, and LS174T (colon cancer), H1299 and H358 (lung cancer), HepG2 (hepatocarcinoma), PC-3 (prostate), THP-1 (acute monocytic leukemia), Daudi (lymphoma), Jurkat (acute T cell leukemia), and MDA-MB-231 (breast cancer), demonstrated that ZZW-115 was efficient to kill these tumor cells with IC_50_ values in the range from 0.42 μM (Hep2G cells) to 7.75 μM (SaOS-2 cells). These data suggest that ZZW-115 could be potentially active for treating cancers from various tissues by targeting NUPR1. Importantly, we have also validated the anticancer effect of ZZW-115 in vivo in both hepatocarcinoma and glioma xenografted tumors (unpublished data).

## 8. ZZW-115 Inhibits the Nuclear Translocation of NUPR1 by Competing with Importins

**Figure 3:**
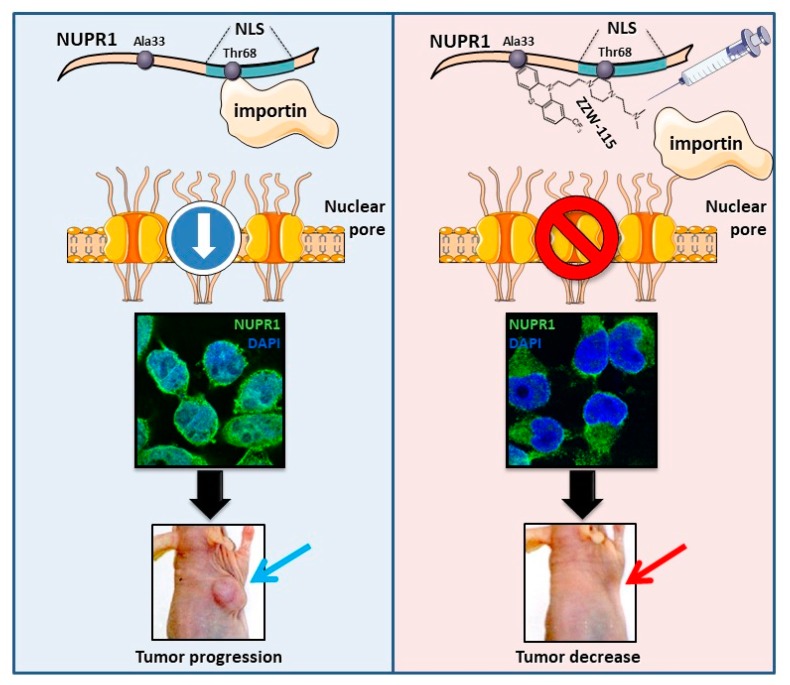
ZZW-115 hampered the nuclear translocation of NUPR1. (Left panel) NUPR1 is a nuclear protein with a predicted NLS. This part of the protein binds to importins and facilitates its translocation from the cytoplasm to the nucleus, through the nuclear pore complex. Thus, NUPR1 exhibits a nuclear localization (as showed in this immunofluorescence), where it can develop its activity and promote tumor progression. (Right panel) Nuclear magnetic resonance data indicated that ZZW-115 binds to NUPR1 in the residue Thr68 [35,37], located within the NLS. Thus, pharmacological inhibition of NUPR1 hampered the interaction with importin and its translocation to the nucleus (as showed in this immunofluorescence). After ZZW-115 treatment, NUPR1 was located in the perinuclear and cytoplasmic area, and inhibition of the nuclear activity of NUPR1 induced tumor growth arrest.

NUPR1 is a nuclear protein that contains a canonical bipartite domain of positively charged amino acids, typical of NLS, involving protein residues 63–78, as tested by theoretical predictions and site directed mutagenesis [40]. The interactome analysis of NUPR1 revealed that it may bind to 30 components of the nuclear pore including several importins or karyopherins (KPNA1, KPNA2, KPNA3, KPNA4, and KPNA6) and 17 NUP proteins (unpublished data). In our previous work, we have shown that NUPR1 binds ZZW-115 by using residues around Ala33 and Thr68 [38], the two hot-spot regions of NUPR1 [35,37]. Because Thr68 belongs to the NLS region of NUPR1, it is probable that ZZW-115 can hinder the interaction between NUPR1 (through its NLS) and importins, and then it can block the NUPR1 nuclear translocation. Therefore, by using NUPR1 immunofluorescence staining, we have studied the potential impact of ZZW-115 on the intracellular location of NUPR1, and we have found that treatment with ZZW-115 inhibits almost completely the translocation of NUPR1 from the cytoplasm to the nucleus (Figure 3) (unpublished results). This result has led us to the conclusion that ZZW-115 can inactivate NUPR1 by preventing its translocation into the nucleus, where it is presumed to play its essential roles regarding cell survival.

## 9. Conclusions

Our work provides a proof-of-concept that stress proteins are effective therapeutic targets for treating cancers. In our work, we have shown how repurposing a drug can be used as a starting point to improve the design and the efficiency of better molecules against cancer, even for challenging targets such as IDPs. Our studies constitute an innovative example of successful ligand-based design (as opposed to structure-based design, employed in the drug-design of well-folded proteins) of an inhibitor for an entirely unfolded protein.
